# Bandgap renormalization and work function tuning in MoSe_2_/hBN/Ru(0001) heterostructures

**DOI:** 10.1038/ncomms13843

**Published:** 2016-12-14

**Authors:** Qiang Zhang, Yuxuan Chen, Chendong Zhang, Chi-Ruei Pan, Mei-Yin Chou, Changgan Zeng, Chih-Kang Shih

**Affiliations:** 1 Department of Physics, University of Texas at Austin, Austin, Texas 78712, USA; 2 Hefei National Laboratory for Physical Sciences at the Microscale (HFNL), CAS Key Laboratory of Strongly-Coupled Quantum Matter Physics, Hefei 230026, China; 3 Department of Physics, University of Science and Technology of China, Hefei 230026, China; 4 School of Physics, Georgia Institute of Technology, Atlanta, Georgia 30332, USA; 5 Institute of Atomic and Molecular Sciences, Academia Sinica, Taipei 10617, Taiwan; 6 Department of Physics, National Taiwan University, Taipei 10617, Taiwan; 7 International Center for Quantum Design of Functional Materials (ICQD), HFNL, University of Science and Technology of China, Hefei 230026, China; 8 Synergetic Innovation Center of Quantum Information and Quantum Physics, University of Science and Technology of China, Hefei 230026, China

## Abstract

The van der Waals interaction in vertical heterostructures made of two-dimensional (2D) materials relaxes the requirement of lattice matching, therefore enabling great design flexibility to tailor novel 2D electronic systems. Here we report the successful growth of MoSe_2_ on single-layer hexagonal boron nitride (hBN) on the Ru(0001) substrate using molecular beam epitaxy. Using scanning tunnelling microscopy and spectroscopy, we found that the quasi-particle bandgap of MoSe_2_ on hBN/Ru is about 0.25 eV smaller than those on graphene or graphite substrates. We attribute this result to the strong interaction between hBN/Ru, which causes residual metallic screening from the substrate. In addition, the electronic structure and the work function of MoSe_2_ are modulated electrostatically with an amplitude of ∼0.13 eV. Most interestingly, this electrostatic modulation is spatially in phase with the Moiré pattern of hBN on Ru(0001) whose surface also exhibits a work function modulation of the same amplitude.

## Abstract (web summary)

Direct epitaxial growth of vertically stacked layered materials is a promising route towards scalable fabrication of van der Waals heterostructures. Here, the authors demonstrate molecular beam epitaxy of semiconducting MoSe_2_ on a hBN/Ru(0001) substrate.

The emergence of semiconducting transition metal dichalcogenides (TMDs) as two-dimensional (2D) electronic materials has triggered intensive research activities^1^,^2^,^3^,^4^. In conjunction with graphene (a semi-metal) and hBN (a large gap insulator), they form a diverse tool set for tailoring novel 2D electronic systems. One particularly powerful approach is stacking different types of van der Waals (vdW) materials to form vdW heterostructures^5^. Many conceptual demonstrations of vdW heterostructures have been achieved by using mechanical exfoliations of vdW layers and then stacking them together using transfer methods^6^,^7^,^8^. This exfoliation/transferring approach, however, is not scalable. An attractive and scalable approach is the direct epitaxial growth of 2D heterostructures, which has recently been shown in several systems using ambient chemical vapour deposition (CVD)^9^,^10^,^11^,^12^,^13^,^14^,^15^. Nevertheless, achieving atomic scale control of contamination using ambient CVD is quite challenging. As an ultra-high-vacuum (UHV)-based growth technique, molecular beam epitaxy (MBE) should provide better control of interface formation^16^, although the number of 2D heterostructure systems demonstrated is more limited^17^,^18^,^19^,^20^,^21^.

Single-layer hBN on transition metal surfaces, including Ru(0001), is a very interesting platform with very rich phenomena^22^,^23^,^24^. It had been used as a platform to grow graphene/hBN heterostructures^25^,^26^,^27^,^28^ and moreover, such heterostructures can be separated from the Ru(0001) substrate by using electrochemical exfoliation^29^,^30^. Owing to the slight lattice mismatch between the hBN and Ru(0001) surfaces, a so-called nanomesh Moiré pattern forms^22^,^23^. Such a Moiré pattern introduces not only height corrugation on hBN (ref. 23) but also periodic modulation in the local work function^31^,^32^,^33^. These properties make the single-layer hBN/Ru(0001) and related systems an ideal platform for investigating how the local work function impacts the electronic structure of the overlayer on top of hBN/Ru(0001) substrate.

In this paper, we report the MBE growth and scanning tunnelling microscopy/spectroscopy (STM/STS) investigation of MoSe_2_ on single-layer hBN on Ru(0001). We not only demonstrate the feasibility of growing MoSe_2_/hBN heterostructure using MBE but also illustrate a convincing case of electronic structure tuning of TMDs. We find the strong interaction between hBN and Ru(0001) makes hBN inseparable from the underlying Ru. The interaction arising from the local atomic registry between the layers and the resulting charge transfer are verified by first-principles calculations. The hBN/Ru(0001) system as a whole has a strong electrostatic screening effect on the band structure of MoSe_2_, reducing the MoSe_2_ quasi-particle gap by 0.25 eV compared with that of MoSe_2_ on graphene or graphite substrates^34^. Furthermore, we show that the local work function modulation on single-layer hBN results in a rigid shift of the band structure of MoSe_2_ without changing the bandgap, indicating a pure electrostatic effect. Moreover, the modulation of the MoSe_2_ band edges is about 0.13 eV, quantitatively similar to the local work function modulation amplitude in hBN. In addition, the work function in MoSe_2_ shows the same modulation, illustrating a convincing case of real-space electrostatic tuning of the band profile.

## Results

### Growth and characterization of MoSe_2_/hBN/Ru(0001)

**Figure 1:**
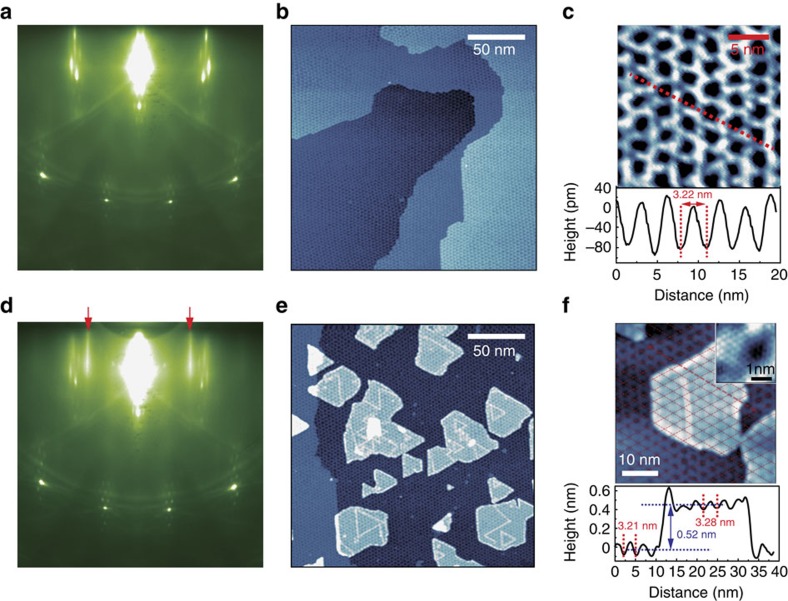
RHEED and STM characterizations of hBN/Ru(0001) and MoSe_2_/hBN/Ru(0001). (**a**) RHEED pattern of epitaxial single-layer hBN on the Ru(0001) substrate. (**b**) Large-scale STM image (−2 V, 5 pA, 215 nm × 215 nm) shows the uniform high-quality single-layer hBN on Ru(0001). (**c**) The upper panel is the high-resolution STM image (−2 V, 5 pA, 20 nm × 20 nm) of the hBN nanomesh (Moiré pattern), and the lower panel is the height profile along the red dashed line. The periodicity and corrugation of the nanomesh are about 3.2 and 0.1 nm, respectively. (**d**) RHEED pattern of the single-layer MoSe_2_ thin film grown on hBN/Ru(0001). The sharp and uniform streaks (indicated by red arrows) reflect the successful synthesis of the MoSe_2_ film. (**e**) Large-scale STM image (−2 V, 5 pA, 215 nm × 215 nm) shows the successful fabrication of nanoscale MoSe_2_ islands. The bright lines in the single-layer MoSe_2_ regions are domain boundaries. (**f**) The top panel is the STM image of a typical MoSe_2_ island, and the inset reveals the atomic resolution of the MoSe_2_ layer. The bottom panel is the height profile along the red dashed line. The corrugation amplitude of the MoSe_2_ surface is about 0.8 Å and is completely in phase with that of the underlying hBN, indicating that the periodic structure on the island is actually a transparent pattern from the nanomesh (corrugation amplitude of ∼1.3 Å) through the single-layer MoSe_2_, instead of a Moiré pattern formed by MoSe_2_ and the underlying hBN.

MoSe_2_/hBN/Ru(0001) heterostructures are synthesized in an all-UHV approach. First, single-layer hBN is prepared on Ru(0001) following the standard UHV-CVD procedure^22^,^23^. Put briefly, hBN forms by the catalytic dehydrogenation of borazine molecules on the Ru(0001) surface at proper vapour pressure and temperature. The high quality of hBN is confirmed by *in situ* reflection high-energy electron diffraction (RHEED) and STM. Shown in Fig. 1a is the RHEED pattern after the hBN growth on Ru(0001), with sharp spots indicating perfect crystallinity of the sample surface. Note that there are six dots arranged at hexagon corners surrounding each bright spot on the first Laue ring, reflecting the existence of a Moiré pattern. Figure 1b shows a typical large-scale STM image of continuous single-domain hBN. A nanomesh Moiré pattern is clearly seen. The full coverage of single-domain hBN observed here agrees with the spotty characteristic of RHEED. After a full coverage of single-layer hBN, additional exposure to borazine molecule would not lead to additional growth suggesting that borazine molecules no longer have access to the catalytic Ru(0001) surface. The zoomed-in image of the nanomesh shown in Fig. 1c reveals that the periodicity of the nanomesh pattern is 3.2 nm, in agreement with the periodicities of 13 × 13 hBN and 12 × 12 Ru(0001) lattices^23^,^35^. Meanwhile, two distinct topography features on the nanomesh are seen: the lower and strongly bound regions assigned as holes; and the higher and loosely bound regions assigned as wires. This uneven binding causes the corrugation of the hBN, with an average amplitude of about 0.1 nm.

After confirming the high quality of hBN/Ru(0001), we transferred this sample *in situ* to the MBE system for MoSe_2_ growth. This all-UHV approach produces a heterostructure with a clean and sharp interface. Additional sharp and uniform RHEED streaks (indicated by red arrows) in Fig. 1d reflect the successful formation of flat crystalline MoSe_2_ layers. In Fig. 1e, the large-scale STM image shows MoSe_2_ islands with diameters from tens to hundreds of nanometres and the bright clusters in the centre of islands are double-layer MoSe_2_. The apparent sharp lines within the MoSe_2_ are domain boundaries, which looks brighter because of the extra finite local density of states (LDOS) in the bandgap^36^,^37^. In Fig. 1f, the top panel shows a typical MoSe_2_ island, the inset shows an atomic resolution image taken from single-layer MoSe_2_, and the lower panel displays the height profile along the red dashed line. From the grid in the upper panel and the height profile in the lower panel, it is evident that the superstructure of the MoSe_2_ island has the same periodicity, corrugated amplitude and phase as the underlying hBN. Surprisingly, the expected Moiré pattern of MoSe_2_ and hBN, whose periodicity is ∼1 nm, is not observed here. Rather the superstructure visible on the MoSe_2_ is just a replication of the Moiré pattern from the underlying hBN, evident by the match in phase and periodicity of spatial modulation.

### Bandgap renormalization of MoSe_2_/hBN/Ru(0001)

The renormalization of the bandgap in 2D materials due to the substrate is an intriguing concept that receives a lot of attentions recently^10^,^17^,^34^,^38^,^39^. It has been shown that single-layer MoS_2_ grown on Au(111) has a significantly lower bandgap than that grown on graphite^10^,^38^,^39^. In the case of MoSe_2_, earlier study suggested that MoSe_2_ on graphite has a smaller gap than MoSe_2_ on bilayer graphene^17^. However, later investigations indicated that the two cases have identical bandgaps^34^. The availability of hBN/Ru(0001) as the substrate that provides an excellent opportunity to revisit this important issue because hBN is a large gap insulator, which is anticipated to provide better isolation of the screening effect.

**Figure 2:**
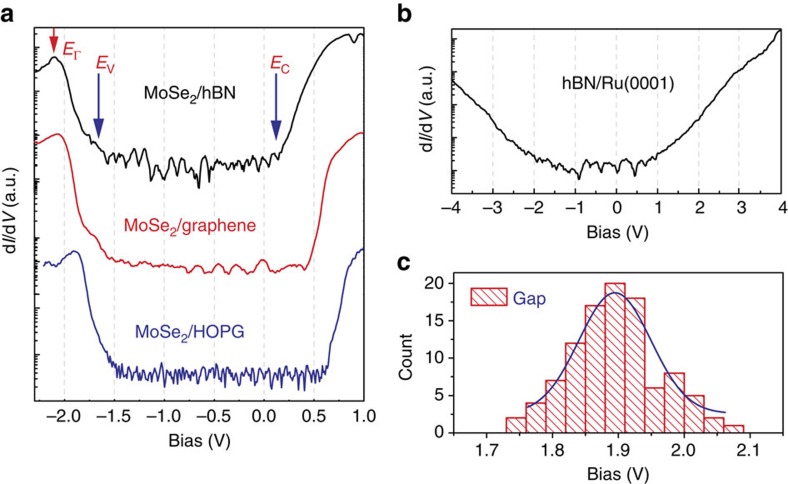
Tunnelling spectroscopy of MoSe_2_ and hBN/Ru(0001). (**a**) Logarithm of d*I*/d*V* spectra for single-layer MoSe_2_ grown on different substrates. MoSe_2_/hBN/Ru(0001), MoSe_2_/graphene/SiC and MoSe_2_/HOPG are shown in black, red and blue, respectively. The MoSe_2_ layer on hBN has a smaller quasi-particle bandgap (by about 0.25 eV) than that of the MoSe_2_ on graphene or graphite substrates. (**b**) Logarithmic d*I*/d*V* of hBN/Ru(0001) shows the remnant metallic characteristics, due to the strong interaction between single-layer hBN and Ru(0001). (**c**) Statistical distribution (from 102 individual d*I*/d*V* spectra) of the quasi-particle bandgap measured for MoSe_2_/hBN. Mean value: 1.90 eV; s.d.: 0.07 eV.

In Fig. 2a we show tunnelling conductance spectra with a fixed tip–sample distance, denoted as d*I*/d*V*, of MoSe_2_/hBN/Ru(0001), together with that of MoSe_2_/HOPG and MoSe_2_/graphene/SiC for comparison. In these spectra, one can identify positions of the valence band state at the Γ point (labelled as *E*_Γ_), the valence band maximum (VBM; at *K* point) *E*_V_ (located at ∼0.4 eV above *E*_Γ_) and the conduction band minimum (CBM) *E*_C_. The quasi-particle bandgap is the energy difference between *E*_V_ and *E*_C_. The spectra show that single-layer MoSe_2_ on graphene or on HOPG have a similar quasi-particle bandgap of 2.15 eV while single-layer MoSe_2_ on hBN/Ru(0001) has a smaller quasi-particle bandgap of 1.90 eV. The results show that the quasi-particle bandgap of single-layer MoSe_2_ indeed depends on the supporting substrate. Nevertheless, how it is renormalized does not follow the intuition that hBN should provide a better electronic isolation of MoSe_2_ from the substrate. Also shown in Fig. 2b is the tunnelling spectrum acquired on hBN with a relatively large sample stabilization voltage of −4 V (implying a relatively large sample-to-tip distance). Interestingly, significant conductance is still present in the expected gap region of hBN, reflecting remnant metallic characteristics. In Fig. 2c, we show statistical distributions of the results from 102 individual tunnelling spectra measured from different locations. The mean value and the s.d. of the quasi-particle bandgap of the single-layer MoSe_2_/hBN/Ru(0001) is 1.90±0.07 eV. Moreover, the d*I*/d*V* spectrum taken on top of a double-layer MoSe_2_ also shows the bandgap is about 1.58 eV (Supplementary Fig. 1), smaller than the gap of double-layer MoSe_2_ on graphene by 0.22 eV (ref. 34).

Thus, our study convincingly demonstrates the concept of band structure renormalization in TMDs. Nevertheless, the actual manifestation of renormalization is probably more complex than an intuitive interpretation of the substrate electrostatic screening and is discussed further below.

### Work function modulation of MoSe_2_/hBN/Ru(0001)

**Figure 3:**
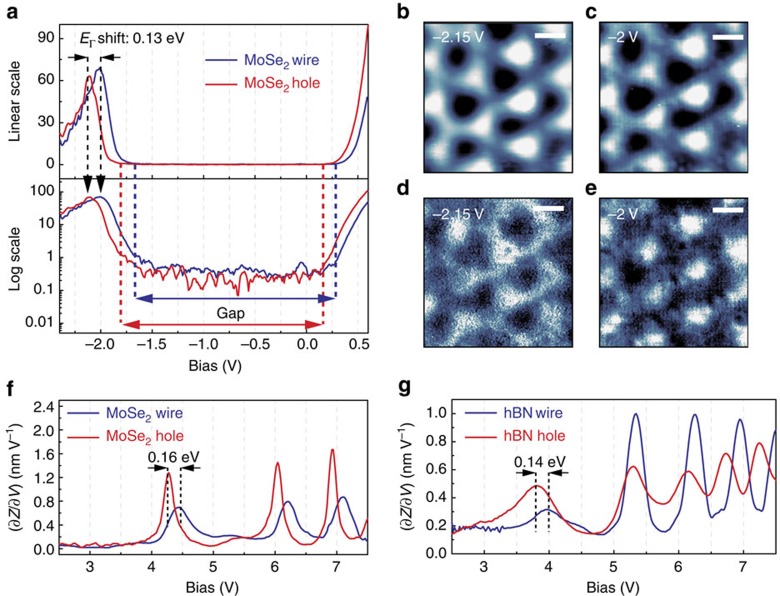
STM images and the tunnelling spectra of MoSe_2_ taken from hole and wire locations. (**a**) The d*I*/d*V* spectra taken on the single-layer MoSe_2_ flake. The tunnelling conductance d*I*/d*V* (with arbitrary unit) is plotted in both the linear scale (upper panel) and the logarithmic scale (lower panel). The black dashed arrows indicate the energy locations of the Γ points, and we observed a rigid shift of the whole band structures (by about 0.13 eV) on hole and wire locations. (**b**,**c**) Topography for the corrugated single-layer MoSe_2_. (**d**,**e**) Corresponding d*I*/d*V* images for **b** and **c**, respectively. Scale bar, 2 nm. (**f**) Field emission resonance (FER) spectroscopy measured on the MoSe_2_ wire (blue) and MoSe_2_ hole (red). (**g**) FER spectroscopy measured on the hBN wire (blue) and hBN hole (red).

Besides the bandgap renormalization discussed above, we have also observed a periodic modulation of the band profile, which is associated with the work function modulation of the nanomesh Moiré pattern. Location-specific STS measurements are shown in Fig. 3. The typical d*I*/d*V* spectra taken from MoSe_2_ hole (red curves) and wire (blue curves) regions are plotted in both the linear scale (upper panel) and the logarithmic scale (lower panel) in Fig. 3a. We use the d*I*/d*V* spectra in the logarithmic scale to determine the CBM, the VBM and the energy of the Γ point in the valance band, Γ_V_ (or *E*_Γ_). While the bandgap values and the energy differences between Γ_V_ and the CBM are the same for both the hole and wire regions, there is a rigid offset for the absolute values of the CBM, VBM and Γ_V_. Such an offset in the band structure is illustrated more drastically in d*I*/d*V* mappings carried out at different tip–sample bias. Figure 3b,c is the topography of the same area at −2.15 and −2.0 V, which are close to Γ_V_ of holes (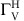) and wires (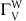), respectively, and they look the same. However, the corresponding d*I*/d*V* mappings taken simultaneously in Fig. 3e,f have a completely reversed contrast; at −2.15 V the wire regions, which are brighter in the topography images, have a lower LDOS, while at −2 V the hole regions, darker in topography, have lower LDOS. This phenomenon confirms the existence of the modulation of the MoSe_2_ band structure. An alternative spectroscopy technique, tip-to-sample distance *Z* versus bias sweep at constant current mode, is used to determine the Γ point values. The (∂*Z*/∂*V*)_*I*_ spectra taken over an ensemble of 120 holes and wires (Supplementary Fig. 2) statistically determine that 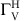
=−2.14±0.03 eV, 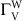
=−2.01±0.02 eV and therefore the amplitude of the periodic modulation of band profile is 0.13±0.05 eV.

In addition, the (∂*Z*/∂*V*)_*I*_ spectra in the field emission region are used, and the sample bias for the first field emission resonance (FER) peak is considered a good approximation of the work function of the sample^32^,^33^. Figure 3f,g shows the local work function modulation of hBN/Ru(0001) is 0.14 eV, and for the MoSe_2_ overlayer such modulation is 0.16 eV, both in excellent agreement with the periodic band offset observed on MoSe_2_. From this consistency, one can conclude that the band offset in MoSe_2_ is purely an electrostatic effect.

## Discussion

**Figure 4:**
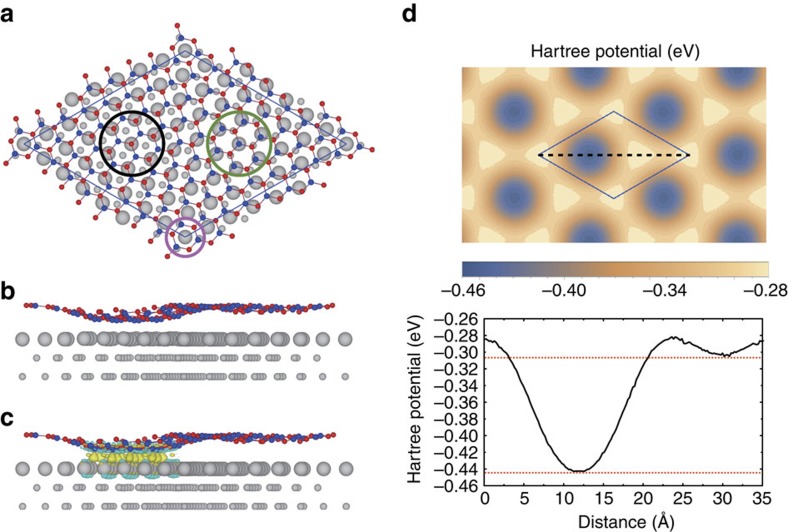
First-principles calculations for the electronic structures of hBN/Ru(0001). (**a**) Top view and (**b**) side view of the atomic structure of √67 × √67 hBN on √57 × √57 Ru(0001). The red, blue and grey spheres are N, B and Ru atoms, respectively. The black, pink and green circles indicate the regions with N atoms at the top, face-centred cubic and hexagonal close-packed sites, respectively, with respect to the Ru(0001) substrate. The average distance between the hBN and the surface Ru layers is about 3.73 Å in the regions indicated by both the green and pink circles and 2.14 Å in the region of the black circle, with a maximal height difference of about 1.67 Å for the hBN layer. (**c**) Charge density difference induced by the interaction, with the yellow (blue) iso-surfaces indicating an increase (decrease) in the charge density. (**d**) Calculated electrostatic potential variations at the height of 4.9 Å above the hBN layer, which corresponds to average of the interlayer distances of hBN and MoSe_2_. The potential at the centre of the vacuum region is set at zero. The profile along the black dashed line is also shown below in which the upper (lower) red dashed line indicates the minimum at the wire (hole) region.

The bonding and resulting change in the electronic structure of hBN on Ru(0001) can be understood by first-principles calculations. The observed Moiré pattern in hBN/Ru(0001) corresponds to roughly 13 × 13 hBN on 12 × 12 Ru(0001), which is too large for a thorough theoretical analysis using plane waves. Instead, we used a supercell with √67 × √67 hBN on √57 × √57 Ru(0001) and a small rotational angle of 5.6°, as shown in Fig. 4a, which provides the essential collection of different atomic registries between the layers and reliable electronic properties for them because the strain (0.45%) is very small. The relaxed atomic configuration is shown in Fig. 4b, indicating that a certain portion of the hBN layer is moved closer to the Ru substrate. This happens in the region near the black circle in Fig. 4a, in which the N atoms are approximately located right above the Ru atoms and strong bonding occurs, giving rise to a calculated interlayer reduction as large as 1.6 Å, in agreement with calculated results obtained previously with different supercells, basis sets and exchange-correlation functionals^40^,^41^,^42^. The bonding can be seen by the iso-surfaces of charge transfer shown in Fig. 4c. In contrast, the green and purple circles mark the regions in which Ru atoms are located right below the B atoms and the centre of the B–N hexagons, respectively. The interlayer interaction is weak, and these two regions are at about 3.7 Å above the Ru plane, a reasonable distance for the vdW interaction.

If we evaluate the electrostatic potential at 4.9 Å above the hBN layer (the average of the vdW layer separations of hBN and MoSe_2_), the value in the hole region is clearly lower than that in the wire region by 0.14 eV, as shown in Fig. 4d. This is in excellent agreement with the relative band edge shift of single-layer MoSe_2_ between the two regions as observed in the experiment. The projected density of states shown in Supplementary Fig. 3 confirms that the significant interaction between hBN and the Ru substrate in the hole region induces states in the hBN gap, giving rise to a metallic characteristic.

Our observation of a bandgap reduction of 0.25 eV for MoSe_2_ on hBN/Ru(0001) proposes interesting possibilities of bandgap renormalization in 2D materials. One probable origin is the extra screening by the states in the gap of hBN arising from the strong interaction with the substrate metal. Keeping this remnant screening effect in mind, one still need to reconcile with the fact that the wire region has a weaker interaction and thus should have a weaker screening (relative to the hole region). Yet we do not observe any difference in the electronic structures between the hole and wire region, except for the electrostatic modulation. One possibility is that the corrugation of the Moiré pattern observed on MoSe_2_ is smaller than that corrugation of hBN Moiré pattern by about 0.5 Å (Fig. 1f), which suggests a modulation of vdW gap distance by the same amount (smaller vdW gap in the wire region). Such a vdW gap modulation would provide a compensation effect. Nevertheless, STM topography does not necessarily reflect the structural contrast quantitatively and thus this explanation is tentative at best. Another possible reason is the significant corrugation (about 0.08 nm) of single-layer MoSe_2_ as shown in Fig. 1f. In planar single-layer MoSe_2_ the VBM (CBM) is the *d*_*xy*_ and 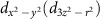 orbitals from Mo. Any local distortion away from the perfect flatness will break the planar symmetry and induce additional hybridization between these *d* orbitals. A bandgap reduction is an entirely plausible result in this situation.

In summary, we have demonstrated the successful MBE growth of single-layer MoSe_2_ islands on top of hBN/Ru(0001). Our STM/STS results have revealed that MoSe_2_ on the strongly coupled hBN/Ru(0001) has a quasi-particle bandgap of 1.90±0.07 eV, 0.25 eV smaller than the results on graphite and graphene. These results on the one hand affirm the concept of band structure renormalization due to the substrate, but on the other hand shows that the renormalization is far more complex than a simple consideration of the metallicity of the substrate and call for more thorough theoretical/experimental investigations. In addition, we show that the local work function modulation on the hBN/Ru(0001) nanomesh structure creates a periodic template of potential modulation, where the band profile of the MoSe_2_ mimics this potential modulation precisely.

## Methods

### Growth of MoSe_2_/hBN/Ru(0001) heterostructures

The clean Ru(0001) surface was obtained on a piece of Ru single crystal by multiple cycles of Xe^+^ sputtering and annealing at 1,000 °C. Hexagonal boron nitride formed on Ru when the single crystal was heated to 1,000 °C and exposed to a borazine vapour of 1 × 10^−6^ torr for about 5 min, then the sample was slowly cooled down to room temperature. The quality of the hBN was checked by STM and RHEED. Then this hBN/Ru(0001) sample was transferred *in situ* to a MBE chamber for the MoSe_2_ growth. The high-purity Mo (99.95%) and Se (99.999%) elemental sources were evaporated from an e-beam evaporator and an effusion cell, respectively, with a ratio of 1:30. Single-layer MoSe_2_ with a coverage of about 30% formed after 45 min of deposition with a substrate temperature of 420 °C, followed by 30 min of annealing at the same temperature with the Se flux maintained.

### Scanning tunnelling microscopy and spectroscopy

All STM investigations reported here were acquired at 77 K in UHV (base pressure is better than 6 × 10^−11^ mbar). A tungsten tip was used. The bias voltage was applied to the sample. The conventional STS, d*I/*d*V*, was acquired at a constant tip-to sample distance (*Z*) with the feedback loop off. The (∂*Z*/∂*V*)_*I*_ spectrum was acquired when the tip-to-sample distance *Z* changes corresponding to the scanning of bias *V* to keep the constant current.

### First-principles calculations

We have performed first-principles calculations with density functional theory as implemented in the Vienna Ab initio Simulation Package^43^. We used the projector augmented wave method^44^ to treat core electrons, and the Perdew–Burke–Ernzerhof (PBE) form^45^ for the exchange-correlation functional with a plane-wave cutoff energy of 300 eV. The periodic slabs contain three Ru layers as the substrate and a vacuum region of about 13 Å. The bottom of the three Ru layers is fixed, while the rest two Ru layers and the hBN layer are allowed to relax during the geometry optimization.

### Data availability

The data that support the findings of this study are available from the corresponding author on request.

## Additional information

**How to cite this article:** Zhang, Q. *et al*. Bandgap renormalization and work function tuning in MoSe_2_/hBN/Ru(0001) heterostructures. *Nat. Commun.* **7,** 13843 doi: 10.1038/ncomms13843 (2016).

**Publisher's note**: Springer Nature remains neutral with regard to jurisdictional claims in published maps and institutional affiliations.

## Supplementary Material

Supplementary Information Supplementary Figures and Supplementary References
