# Supplementary material for: Bandgap renormalization and work function tuning in MoSe2/hBN/Ru(0001) heterostructures
Source: Nat Commun. 2016 Dec 14;7:13843. doi: 10.1038/ncomms13843 (PMC5171833; doi:10.1038/ncomms13843)
Supplement: Supplementary Information — Supplementary Figures and Supplementary References [file ncomms13843-s1.pdf]

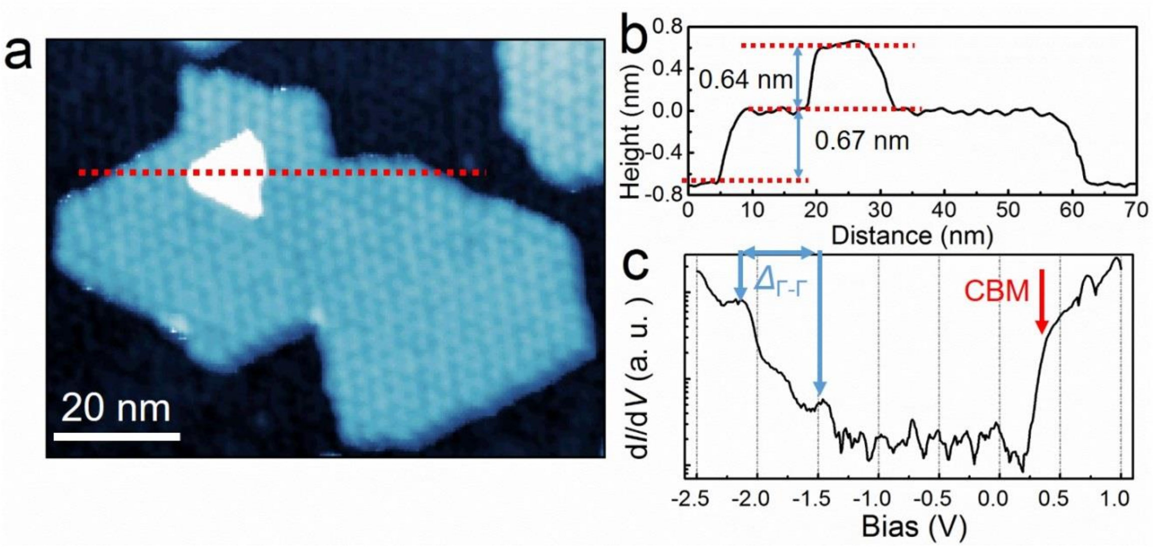

Supplementary Figure 1. STM image and spectrum of DL-$MoSe_2$. (a) STM image of a relatively small DL-$MoSe_2$ on top of a large single-layer $MoSe_2$. (V_b=3 V, I_t=5 pA, 89 nm × 69 nm) **(b)** Height profile along the red dashed line in **(a)**. The apparent heights of SL-$MoSe_2$ and the second layer $MoSe_2$ are 0.64 nm and 0.67 nm, respectively. **(c)** d*I*/d*V* spectrum in logarithm scale indicates the $\Delta_{\Gamma-\Gamma}$ splitting equals to 0.68 eV for the DL-$MoSe_2$.

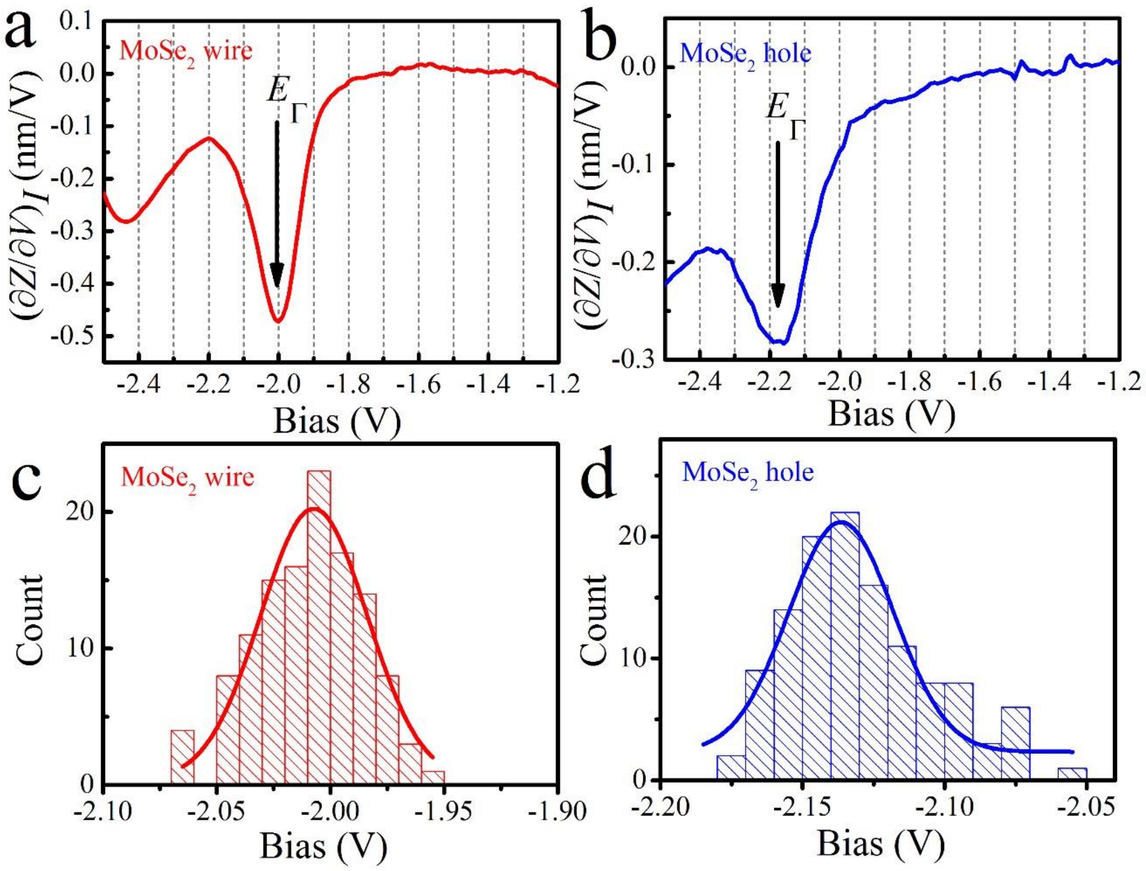

Supplementary Figure 2. ($\partial Z/\partial V$)$_I$ spectra and statistical distributions of the Γ points. **(a,b)** Individual (∂Z/∂V)$_I$ spectra taken from $MoSe_2$ hole and $MoSe_2$ wire locations, respectively. The black arrows indicated the energy locations of the Γ points[1,2]. **(c,d)** Statistical distributions for Γ points of wire and hole, respectively (based on 120 individual ($\partial Z/\partial V$)$_I$ spectra). Γ_V^H = -2.14 ± 0.03 eV, Γ_V^W = -2.01 ± 0.02 eV

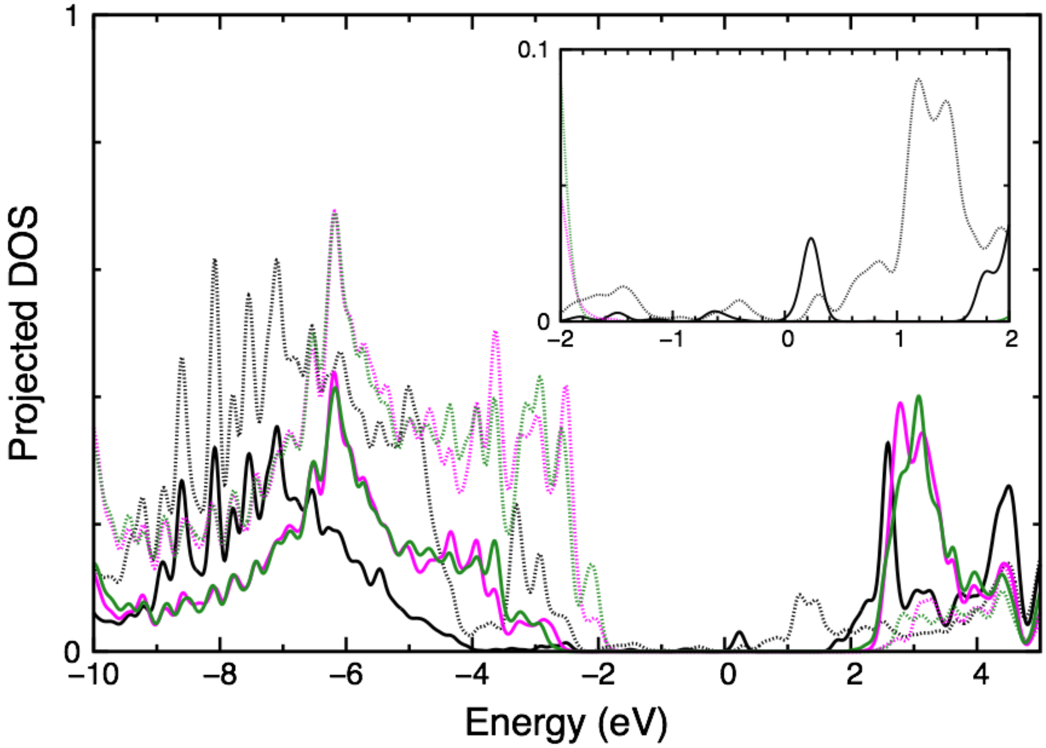

Supplementary Figure 3. Projected density of states on the p orbitals of B (solid lines) and N (dotted lines) atoms in the regions indicated by the black, green, and purple circles in Figure 4a. The Fermi level is set as the zero energy.

Supplementary References

1. Zhang, C. et al. Probing Critical Point Energies of Transition Metal Dichalcogenides: Surprising Indirect Gap of Single Layer WSe2. *Nano Lett.* **15**, 6494-6500 (2015).
2. Zhang, C. et al. Visualizing band offsets and edge states in bilayer-monolayer transition metal dichalcogenides lateral heterojunction. *Nat. Commun.* **7**, 10349 (2016).
